# Evaluating the Influence of MCAT Scores on Medical Student Selection and Performance Using a Machine Learning Approach: Retrospective Cohort Study

**DOI:** 10.2196/94197

**Published:** 2026-09-01

**Authors:** Michele Cherfane, Marc Ghanem, Georges Choueiry, Selim M Nasser, Tamina Elias-Rizk, Sola Aoun Bahous

**Affiliations:** 1 Gilbert and Rose-Marie Chagoury School of Medicine, Lebanese American University, Blat, PO box 36 Byblos, Byblos, Byblos, 4501, Lebanon, +961 1 786456 ext. 2336; 2 Lebanese American University Medical Center-Rizk Hospital, Beirut, Lebanon

**Keywords:** Medical College Admission Test, MCAT, machine learning, medical school admission, medical school performance, student selection

## Abstract

**Background:**

The Medical College Admission Test (MCAT) has been central to medical school admissions in North America, though its necessity in holistic selection processes remains debated. The COVID-19 pandemic’s suspension of MCAT testing sessions allowed institutions to explore alternative admission criteria. Additionally, global challenges in test administration underscore the vulnerability of systems dependent on a single standardized test.

**Objective:**

This study aimed to (1) assess whether including MCAT scores in admissions decisions improves the prediction of medical school performance compared with grade point average (GPA) and interview-based selection and (2) evaluate whether machine learning (ML) can generate viable MCAT score predictions when testing is unavailable.

**Methods:**

We conducted a retrospective cohort study of 1898 applicants to the Lebanese American University School of Medicine (2009‐2023). Among 349 admitted students with MCAT scores, we compared admission composites including vs excluding the MCAT in relation to academic outcomes (Med 1‐4 final grades) and clinical performance (Med 1‐2 objective structured clinical examination [OSCE] scores). Students were stratified into tertiles to examine tier-specific effects. To model an MCAT alternative, we trained an ensemble ML model (least absolute shrinkage and selection operator, kernel ridge, gradient boosting, elastic net, and light gradient boosting machine [LightGBM]) using data from 1583 applicants (2009‐2020) to predict MCAT scores from cumulative GPA, core GPA, interview scores, merit points, and honors participation. The model was validated on 315 applicants admitted during the MCAT suspension (2021‐2023), and its impact on admission rankings and performance correlations was evaluated.

**Results:**

Including the MCAT in the admissions composite did not meaningfully improve prediction of overall academic performance (*r*=0.63, 95% CI 0.56-0.69 with MCAT vs *r*=0.62, 95% CI 0.55-0.68 without MCAT; *P*=.63). However, it significantly weakened prediction of clinical skills (OSCE: *r*=0.37, 95% CI 0.27-0.46 with MCAT vs *r*=0.48, 95% CI 0.40-0.56 without MCAT; *P*<.001). In tertile analyses, the MCAT modestly improved prediction among top-performing students but eliminated predictive validity in the bottom tertile (*r*=0.12, 95% CI –0.07 to 0.30; *P*=.21 vs *r*=0.28, 95% CI 0.10-0.44 without MCAT; *P*=.003). The ML model explained 36% of MCAT variance (*R*²=0.36 with 95% CI 0.32-0.41; root mean squared error ≈10% of score range). Incorporating predicted MCAT scores reduced correlations with subsequent performance across all outcomes. Although rankings based on predicted MCAT scores strongly correlated with original rankings (*r*=0.80, 95% CI 0.77-0.83), 6.3% (4/64) to 9.4% (6/64) of admission decisions would have changed.

**Conclusions:**

Within this single-institution study, MCAT scores provided limited incremental validity for predicting academic performance and reduced prediction of clinical skills. ML-based predictions of MCAT scores introduced sufficient error to affect admissions decisions, supporting a robust holistic admissions process during temporary MCAT disruptions while highlighting the need for validation across diverse institutional settings before broader generalization.

## Introduction

### Background

Selecting future medical students is a high-stakes endeavor that shapes the future physician workforce. Admissions committees strive for a holistic review that balances academic excellence with personal attributes. In North American models, this means combining metrics like undergraduate grade point average (uGPA) and Medical College Admission Test (MCAT) scores with evaluations of nonacademic qualities such as interview performance and situational judgment tests [1-4]. This multifactor approach aims to identify well-rounded candidates who possess the knowledge, skills, and character to succeed as physicians.

The MCAT has long held a central role as a standardized measure of applicants’ academic preparedness. Since its introduction, the examination has evolved from a predominantly knowledge-based assessment into a competency-oriented tool designed to evaluate scientific reasoning, critical analysis, and understanding of behavioral and social determinants of health [5]. This evolution reflects broader shifts in medical education toward competency-based training and more holistic admissions practices. Several studies evaluating the newer versions of the MCAT have demonstrated its predictive validity, particularly for student performance in the first year of medical school, and, to a lesser extent, throughout subsequent stages of training [4,6-9]. Despite this, debate has persisted within the medical education community regarding whether the exam carries disproportionate weight in admissions decisions, with some advocating for its reduced emphasis or elimination [10].

These debates extend beyond medical education and reflect broader discussions regarding standardized testing in higher education. Supporters of standardized examinations argue that they provide a common benchmark across heterogeneous educational systems and help ensure comparability among applicants from diverse academic backgrounds [11,12]. Critics, however, have raised concerns that excessive reliance on test-based selection may unintentionally reinforce socioeconomic and educational inequities, particularly given unequal access to preparatory resources, variations in educational opportunity, and differential application patterns across socioeconomic groups [13]. Recent national data from MCAT examinees further demonstrate persistent socioeconomic disparities in examination performance and the likelihood of applying to medical school, reinforcing concerns regarding equity in admissions [14]. These debates have also been informed by broader psychometric phenomena, including the Flynn effect, which describes the long-term increase in standardized cognitive test scores across generations, estimated at approximately 2 to 3 IQ points per decade across many populations, and highlights that population-level changes may influence the interpretation and ongoing evaluation of standardized assessments [15,16]. Consequently, many medical schools have increasingly adopted holistic review approaches that seek to balance cognitive metrics with assessment of interpersonal, ethical, and professional attributes [17].

Importantly, the North American medical admissions model differs substantially from that used in many other regions of the world. While many countries admit students directly into medical education following secondary school, North American institutions generally require completion of a prior undergraduate degree and evaluate applicants through multiple layers of academic and nonacademic assessment. Within this framework, the MCAT serves not only as a measure of academic preparedness but also as a standardized comparator across diverse undergraduate institutions and educational pathways [12]. At the same time, the growing use of situational judgment tests and structured interviews reflects increasing recognition that no single examination fully captures the competencies required for future physicians [17,18].

### Admissions During the COVID-19 Pandemic

The widespread suspension of the MCAT exam during the COVID-19 pandemic created a unique opportunity that allowed institutions to explore alternative admissions criteria, relying more heavily on grade point average (GPA) and interviews [19]. While GPA is a consistent academic predictor, interview scores have been shown to be less reliable and predictive of success [20-25], may unintentionally favor some demographic groups [26], and can be influenced by applicants’ prior academic performance [27]. Beyond the pandemic, global challenges in MCAT administration, ranging from limited test center capacity to policy restrictions, such as the elimination of the examination in Quebec following new language requirements, underscore the vulnerability of admissions systems that depend heavily on a single standardized test. These experiences encouraged investigation into innovative approaches, such as predictive modeling, that could support admissions decision-making when standardized testing is disrupted.

In Lebanon, the MCAT was suspended for 2 consecutive years due to the pandemic, requiring the Lebanese American University’s (LAUs) medical school admissions to rely solely on interview scores and uGPA. This experience raised 2 fundamental questions: How essential is the MCAT to making sound admissions decisions, and can technology offer a stand-in for the MCAT when it is unavailable?

On the one hand, if admissions without the MCAT yield similar outcomes, it would suggest that the test, while useful, may be somewhat redundant with other indicators (like GPA). On the other hand, the MCAT might add a unique standardization that, in its absence, could be approximated using predictive modeling. Recent advances in machine learning (ML) make it conceivable to predict an applicant’s likely MCAT score from their academic record and other attributes. If such an ML model is accurate, it could serve as a temporary tool for admissions committees during periods when traditional testing is disrupted (eg, pandemics, natural disasters, or sociopolitical instability). Importantly, framing ML in this context is not to replace the MCAT permanently but to support decision-making when the test cannot be administered.

### Study Objectives

Building on this context, the present study had two prespecified objectives: (1) to assess whether including MCAT scores in admissions scoring improves prediction of medical school performance compared with decisions made without the exam and (2) to evaluate the feasibility of an ML-based model that predicts MCAT scores from other admissions data as a potential alternative when the exam is unavailable.

## Methods

### Overview of the Admission Process

**Figure 1.**
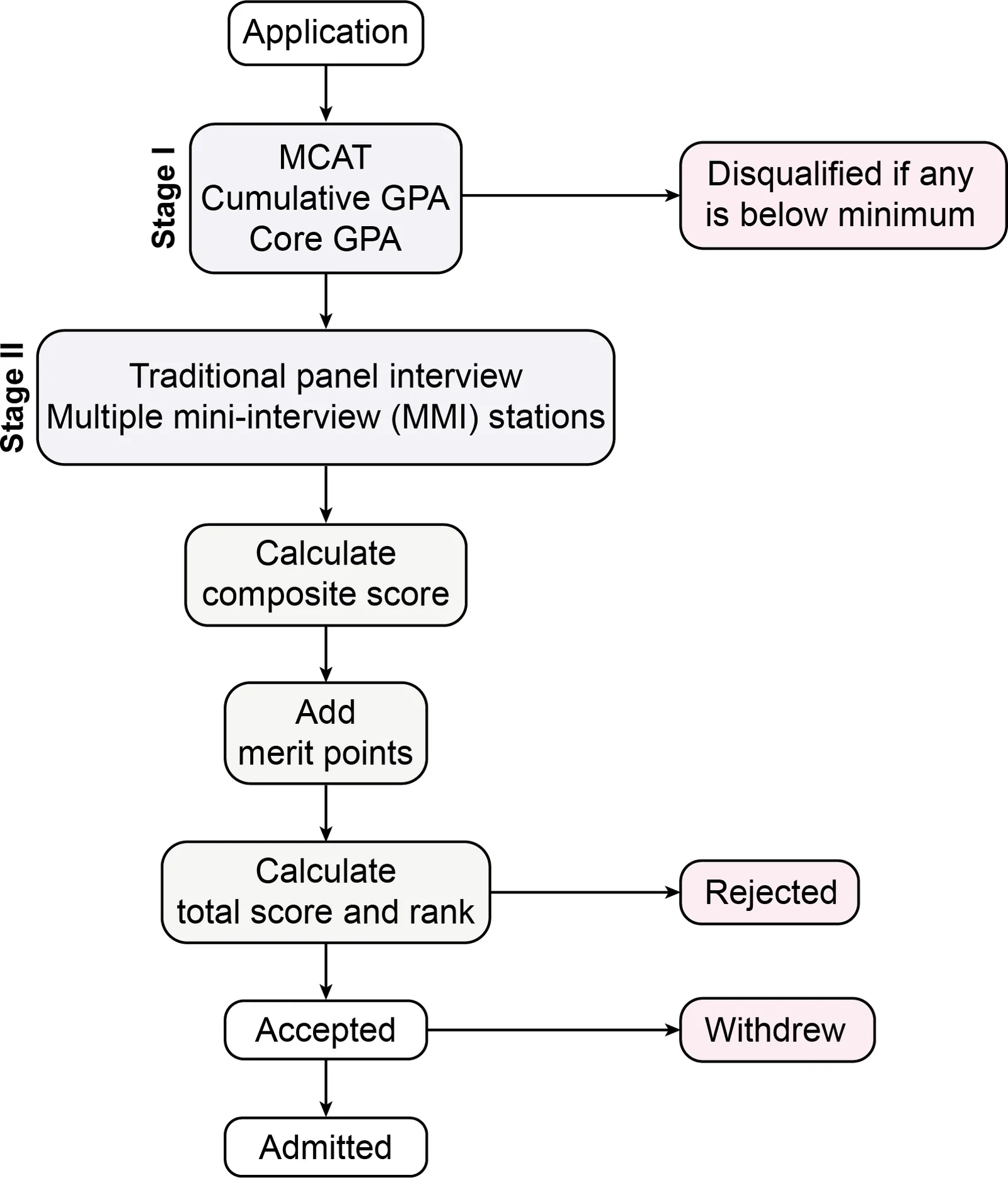
Admission process at the Lebanese American University (LAU) School of Medicine. GPA: grade point average; MCAT: Medical College Admission Test.

The LAU School of Medicine (SOM) follows the American model of medical education and offers a 4-year Doctor of Medicine program. Established in 2009, the SOM welcomed its first cohort the same year, initially enrolling 23 students; class size then increased progressively, in line with university strategy, to 64 students. Admission is conducted through a 2-stage process (Figure 1). In the first stage, applications are reviewed based on academic criteria including MCAT and uGPA scores. The uGPA incorporates both the cumulative GPA and a core GPA calculated from grades in prerequisite courses such as biology, biochemistry, and other disciplines considered essential for medical studies by the school. Applicants must meet specific minimum thresholds for each component (MCAT, cumulative GPA, and core GPA), below which they are automatically disqualified from consideration.

In the second stage, selected candidates are invited for interviews designed to assess nonacademic qualities. These interviews include traditional panel interviews, as well as multiple mini-interview (MMI) stations, which were introduced at the school in 2018. The outcomes from both stages are combined into a final composite score, with weightings determined by the admissions committee. Finally, applicants enrolled in the honors program and those with exceptional talents, research experience, or unique achievements are awarded up to 2 merit points (over 100), which are added to their composite score. Over time, the relative weighting of composite score components has been adjusted through continuous programmatic evaluation of the admissions process.

The admissions committee itself reflects a broad representation from across the university. Membership includes SOM faculty from basic science and clinical departments, faculty from undergraduate programs, as well as medical students, graduates of the SOM, and representatives from various university offices. Admissions decisions follow a structured deliberation process: they begin with a framework developed by admissions leadership that aligns with the school’s mission and institutional priorities, incorporate data from internal validation studies, and undergo iterative discussion and consensus-building among committee members before final approval.

During the 2 academic years when the MCAT was suspended due to the COVID-19 pandemic, the committee maintained the same deliberative process while adjusting the admissions model. In place of the MCAT, the committee adopted an alternative weighting scheme that emphasized uGPA and interview performance. This weighting was based on the collective wisdom and consensus of the committee and subsequently approved through the school’s established procedures for modifying admissions processes. As a result, admissions decisions during this period continued to follow the same rigorous, mission-driven, and data-informed framework used in all other years (Table 1). To ensure consistent implementation, interviewers received standardized guidance and scoring rubrics (adapted for remote delivery where needed), panel composition and scoring protocols were preserved, minimum thresholds for uGPA and core GPA remained unchanged, and merit or honors point allocation followed existing rules; all pandemic-era interview ratings were recorded in the institutional database, and interviewers were asked to flag any connectivity or format issues to support individualized review of borderline applicants.

**Table 1.** Weight distribution of admission composites before and during the Medical College Admission Test (MCAT) suspension.

Component	Cohorts of 2009‐10 to 2020‐21	Cohorts of 2021‐22 and 2022‐23
Cumulative GPA^a^, %	25‐30	40
Core GPA, %	20	35
MCAT, %	30‐35	—^b^
Interview^c^, %	15‐25	25

a GPA: grade point average.

b Not available.

c Includes traditional interviews with multiple mini-interview stations since 2018.

### Study Design and Data Collection

This study is a retrospective cohort analysis of 1898 candidates who submitted complete applications to the Doctor of Medicine program at LAU from 2008 (for the 2009‐2010 academic year, when the program was launched) to 2021 (for the 2022‐2023 academic year). For each applicant, we collected data on cumulative GPA, core GPA, MCAT score (when available), and interview grade. For admitted candidates, additional performance measures were collected, consisting of end-of-year grades for Med 1 through Med 4 and average objective structured clinical examination (OSCE) scores from Med 1 and Med 2. Throughout the study period, the SOM maintained standardized grading procedures, assessment frameworks, and OSCE evaluation methods across cohorts. Although minor curricular refinements occurred over time, the core competency framework and evaluation principles remained consistent.

### Analytical Approach (Aligned with Objectives)

#### Evaluating the Role of the MCAT in Predicting Medical School Performance (Objective 1)

To address objective 1, we recalculated admissions composites with and without the MCAT and compared their associations with subsequent medical school outcomes. In other words, we examined whether the actual admissions model that includes MCAT scores shows a stronger association with subsequent student performance compared with an MCAT-excluded admissions model. For the 349 admitted students in years when the MCAT was administered, we recalculated a hypothetical admissions composite that excluded the MCAT, using the weighting scheme that was adopted by the admissions committee during the COVID-19 suspension period (the right column of Table 1).

This approach generated 2 parallel admission scores for each student: 1 incorporating the MCAT and 1 excluding it. We then tested, using Pearson correlation analysis, the association between each score and subsequent medical school performance, as measured by final-year grades from Med 1 to Med 4 and average OSCE scores from Med 1 and Med 2. We also examined the nonlinear relationship between admission scores (with and without MCAT) and academic outcomes (average OSCE scores and average final-year grades) using generalized additive models with cubic splines. For each outcome, we had 2 models: 1 using the admission score with MCAT and 1 without it. To adjust for overfitting, given our small sample size of 349 admitted students, model performance was evaluated using *R*² obtained from 10-fold cross-validation. The additional value provided by MCAT was determined by calculating the difference between *R*² of the model with MCAT and *R*² of the model without MCAT. The 95% CIs for this difference were calculated using 500 bootstrap resamples.

To examine whether the value of MCAT differs across students’ admission ranks, we tested the interaction between MCAT score and admission rank in a linear regression model with average final-year grade as the outcome, controlling for cumulative GPA, core GPA, interview score, honors program status, and merit score. Because a single interaction term assumes a uniform linear change across ranks, we complemented this analysis by stratifying students into tertiles based on admission composite scores: top third (n=119), middle third (n=117), bottom third (n=113), and recalculated the correlation with the average final-year grade for each tier. This allowed us to determine the predictive value of MCAT specifically for the most critical students in the bottom tier, who are at the greatest academic risk.

#### Developing an ML Model to Predict MCAT (Objective 2)

To address objective 2, we trained and validated an ensemble ML model to predict MCAT scores from preadmission data and assessed the impact of predicted scores on rankings and outcome correlations. For this, we used data from 1583 medical school applicants with available MCAT scores from academic years 2009‐2010 to 2020‐2021 to train an ensemble of 5 models to predict MCAT scores based on the following preadmission information: core GPA, cumulative GPA, interview score, merit points, and participation in honors programs. The predictions from all 5 models were then averaged with equal weights to produce final MCAT score estimates. We then used this model to predict MCAT scores for applicants who did not take the exam due to the COVID-19 pandemic during the 2021 to 2022 (n=167) and 2022 to 2023 (n=148) academic years and who qualified for the 2 stages of admission. These predicted scores were incorporated into the overall admission scoring alongside undergraduate GPA and interview scores (using the prepandemic weighting shown in Table 1) to produce new rankings. We then (1) assessed the agreement between rankings produced with and without the predicted MCAT scores and (2) compared the associations between admission scores and medical school performance metrics, including final-year grades from Med 1 to Med 3 (Med 4 data were not yet available for these cohorts at the time of the study) and average OSCE grades from Med 1 and Med 2.

### Statistical Analysis

To address objective 1 and analyze the impact of MCAT, we calculated the correlation coefficients between each admission score (with and without MCAT) and medical school grades. The difference between these correlations was tested using the Steiger Z test.

To address objective 2 and develop an ML model that predicts the MCAT score, the dataset was split into training (60%), validation (10%), and testing (30%) sets using stratified random sampling to maintain score distribution across sets. The validation set was used to assess model behavior during development and to confirm the equal-weight averaging strategy. Final model performance was evaluated on the independent test set. Missing values were imputed using MissForest, a method capable of handling nonlinear relationships and complex interactions [28]. We used an ensemble of 5 models that combined least absolute shrinkage and selection operator, kernel ridge, gradient boosting, elastic net, and light gradient boosting machine (LightGBM) models. All admission scores were scaled before modeling, as some of the models used are sensitive to the scale of predictors and variables with larger ranges would otherwise dominate the regularization penalty and therefore bias the results. Performance was evaluated using *R*² (proportion of variance explained) and root mean squared error (RMSE, average prediction error on the original MCAT scale after denormalization). Feature importance was assessed through permutation importance analysis, which measures the decrease in model performance when each feature is randomly shuffled. We then used the model to predict MCAT scores for admissions during COVID-19, and therefore produce new rankings based on it, as an alternative to the existing method. Agreement between these 2 admission scoring methods was assessed using the Kendall rank correlation coefficient (τ). All statistical tests were 2-sided, and *P*<.05 was considered the threshold for statistical significance.

Data were collected and aggregated using Python (version 3.13.2), and subsequent analyses were performed in R (version 4.5.1; R Foundation for Statistical Computing).

### Ethical Considerations

This study was deemed exempt from full review by the Lebanese American University Institutional Review Board. All admission and medical school performance information was deidentified prior to analysis. Individual informed consent was not obtained. This was a retrospective study using existing student data routinely collected and maintained by the institution for administrative and academic purposes. No direct contact with students occurred for the purposes of this study. The review board waived the requirement for individual informed consent.

## Results

### Sample Description

**Figure 2.**
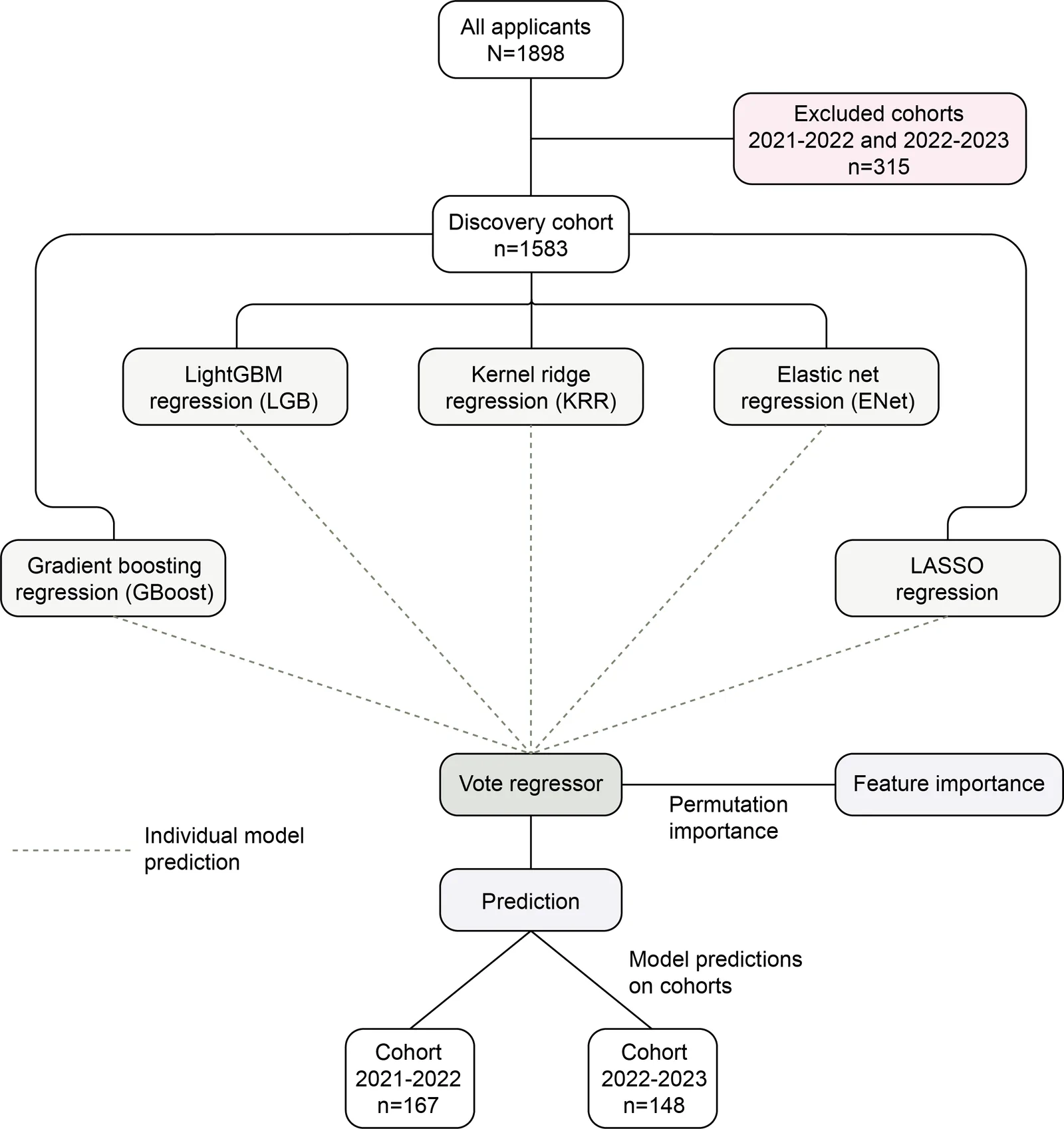
Steps in modeling the Medical College Admission Test (MCAT) score. GBM: gradient-boosting machine; LASSO: least absolute shrinkage and selection operator.

Our sample consists of 1898 candidates with complete applications from 2009 to 2023. The subset used for modeling excludes the last 2 cohorts of 2021 to 2022 and 2022 to 2023, who did not take the MCAT exam due to the COVID-19 pandemic (Figure 2). The description of this subset is presented in Table 2.

**Table 2.** Description of the subset used in modeling Medical College Admission Test (MCAT) scores (N=1583)^a^.

Feature	Values	Missing values
Honors program participation, n (%)		
No	1480 (93.49)	—^b^
Yes	103 (6.51)	—
Merit points, n (%)		
0	1539 (97.22)	—
1	36 (2.27)	—
2	8 (0.51)	—
Scaled interview score (over 100), mean (SD)	81 (11)	678
Cumulative GPA^c^ score (over 100), mean (SD)	51.1 (34)	12
Core GPA score (over 100), mean (SD)	37.3 (36)	26
MCAT score (over 100), mean (SD)	67 (12)	—

a GPA and interview scores were transformed to a common 0‐100 normalized scale for analysis. Therefore, the reported values represent normalized scores rather than conventional GPA values.

b Not applicable.

c GPA: grade point average.

### Impact of Including the MCAT Score in Admissions Decisions on Future Performance (Objective 1)

**Figure 3.**
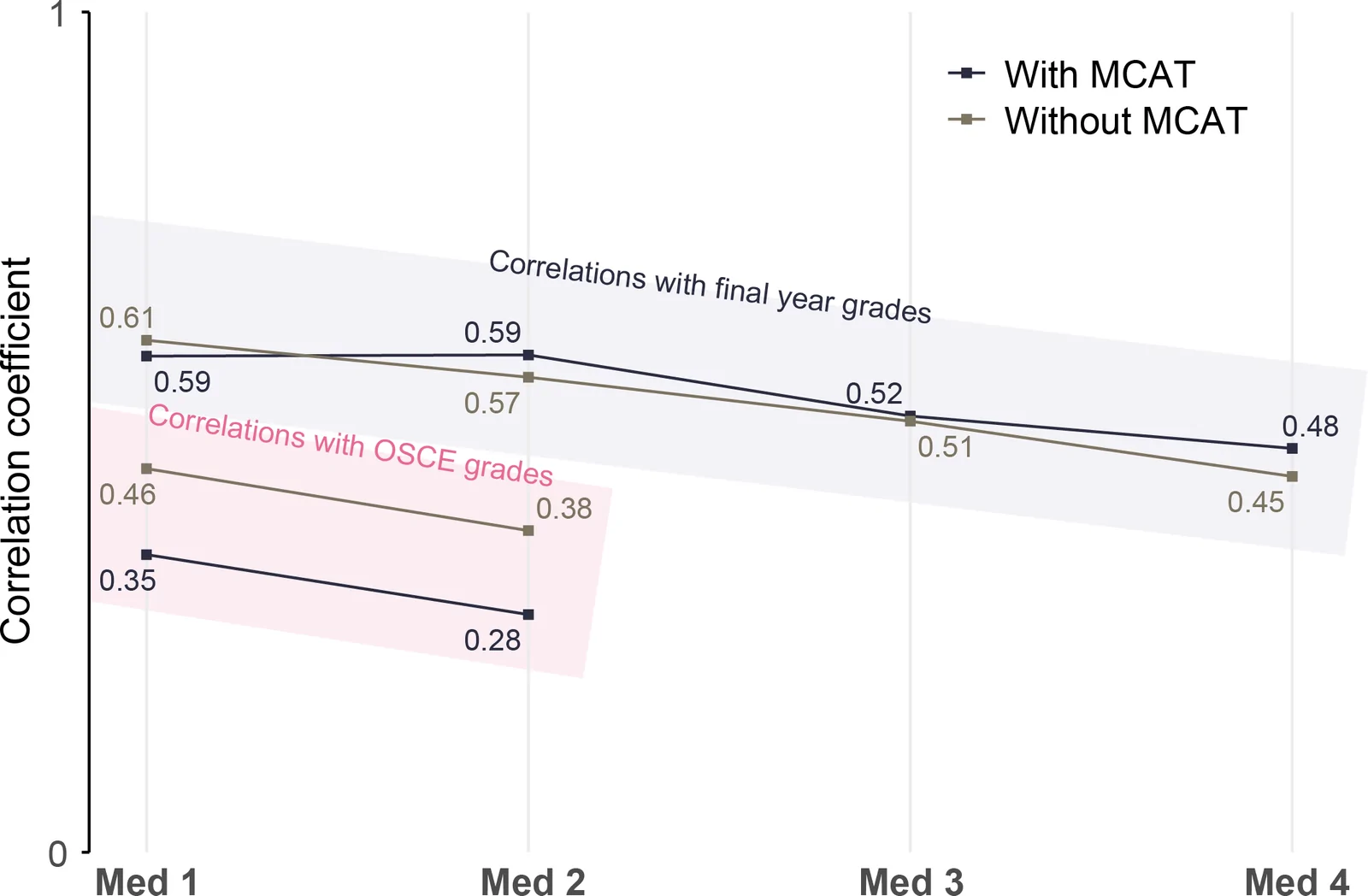
Comparison of the 2 admission models based on the correlation between their scores and grades from Med 1 to Med 4. MCAT: Medical College Admission Test; OSCE: objective structured clinical examination.

Including the MCAT score in the admissions composite did not improve prediction of overall academic performance (n=349 admitted students with MCAT scores and complete outcome data; academic years 2012‐2013 till 2018‐2019). The correlation between the admissions composite score and the average final grade (an aggregate of Med 1‐4 performance) was 0.63 (95% CI 0.56-0.69) when the MCAT was included, compared to 0.62 (95% CI 0.55-0.68) without it, and this difference was not statistically significant (Steiger Z=0.48, *P*=.63). By contrast, for clinical performance as measured by the average OSCE scores from Med 1 and Med 2, the correlation was significantly lower (Steiger Z=−5.35, *P*<.001) when the MCAT was included (*r*=0.37, 95% CI 0.27-0.46) than when it was excluded (*r*=0.48, 95% CI 0.40-0.56). Year-by-year correlations are presented in Figure 3, which shows that this pattern was consistent over time. Nonlinear analysis using generalized additive model was consistent with linear findings. For final-year grades, the difference in *R*² (with MCAT-without MCAT) was Δ*R*²=−0.01 (95% CI −0.04 to 0.03), indicating no meaningful contribution of the MCAT to predictive accuracy. For OSCE grades, the model without MCAT outperformed the model with MCAT, with Δ*R*²=−0.11 (95% CI –0.16 to –0.07), suggesting that including the MCAT in the admission score reduced the prediction of OSCE performance.

After dividing the admitted cohort (n=349) into 3 tertiles based on admission composite scores including MCAT, we recalculated the correlation with the average final-year grade for each tier. In the top third (n=119), the score including MCAT showed stronger correlation than the score without MCAT, with *r*=0.59 (95% CI 0.46-0.69) vs 0.52 (95% CI 0.37-0.64) and both *P*<.001. In the middle tertile (n=117), the correlation was nearly identical whether MCAT was included (*r*=0.33, 95% CI 0.15-0.48; *P*<.001) or excluded (*r*=0.34, 95% CI 0.17-0.49; *P*<.001). Most notably, in the bottom tertile (n=113), the score including MCAT showed no significant correlation with final-year grades (*r*=0.12, 95% CI −0.07 to 0.30; *P*=.21), whereas the score without MCAT maintained a significant small-to-moderate correlation (*r*=0.28, 95% CI 0.10-0.44; *P*=.003). However, interaction between MCAT score and admission rank was not statistically significant in a linear regression model predicting average final-year grade (β coefficient=−0.002, 95% CI −0.004 to <0.001; *P*=.07), although the model may have been underpowered to detect a small interaction effect.

### Modeling an MCAT Alternative and Its Decision Impact (Objective 2)

**Figure 4.**
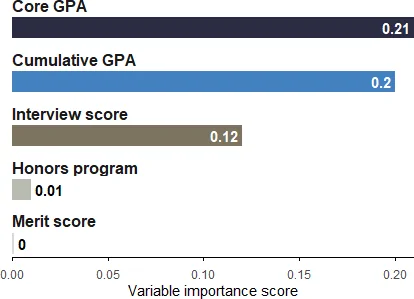
The importance of each predictor in the ensemble model. GPA: grade point average.

We trained an ensemble model to predict the MCAT score using core GPA, cumulative GPA, interview score, merit score, and participation in honors programs. The model achieved an out-of-sample *R*² of 0.36 (95% CI 0.32-0.41) and an RMSE of 0.10 on the normalized scale, meaning it explained 36% of the variability in MCAT scores and predicted them with an average error of 0.1. Since the MCAT score is normalized (ranging from 0-1 in our analysis), this average error of 0.1 corresponds to 10% of the score range (eg, ~5‐6 MCAT points if mapping to 472‐528), which is large enough to reorder applicants near admissions cutoffs. The feature importance plot (Figure 4) shows that core and cumulative GPAs were the most influential factors in predicting the MCAT score, while honors and merit points have little to no effect.

We used the model to predict MCAT scores for the cohorts during which the exam was suspended (2021‐2022 and 2022‐2023) and to calculate new rankings and admission scores using the predicted MCAT. The Kendall rank correlation between the original admission rankings (based solely on GPA and interviews during the MCAT suspension period) and the model-based ranking was 0.80 (95% CI 0.77-0.83; *P*<.001), indicating a strong overall agreement. We found that, had predicted MCAT scores been used, 6 (9.4%) of the 64 admitted students in 2021‐2022 and 4 (6.3%) of 64 in 2022‐2023 would not have been admitted, with an equal number of applicants from the rejected pool selected instead. With respect to merit scholarships, 1 award in 2021‐2022 and 2 awards in 2022 to 2023 would have been reassigned to different students.

However, the new admission scores (with predicted MCAT) showed weaker correlations across all measures of future performance in medical school compared with the actual admission scores from those years (Table 3). This was expected, since the model’s 10% average prediction error introduced significant noise into the new scores.

**Table 3.** Correlation between admission scores, including and excluding predicted modeling of Medical College Admission Test (MCAT) scores and later performance in medical school.

Grade type and medical year	Sample size, n	Original admission score		Model-based admission score	
		Correlation coefficient (95% CI)	*P* value	Correlation coefficient (95% CI)	*P* value
OSCE^a^					
1	129	0.28 (0.11-0.43)	.002	0.24 (0.07-0.40)	.006
2	126	0.33 (0.17-0.48)	<.001	0.27 (0.10-0.43)	.002
Final-year grade					
1	128	0.48 (0.33-0.60)	<.001	0.42 (0.27-0.56)	<.001
2	126	0.46 (0.31-0.59)	<.001	0.39 (0.24-0.53)	<.001
3	63	0.33 (0.09-0.54)	.008	0.23 (-0.01-0.46)	.07

a OSCE: objective structured clinical examination.

**Figure 5.**
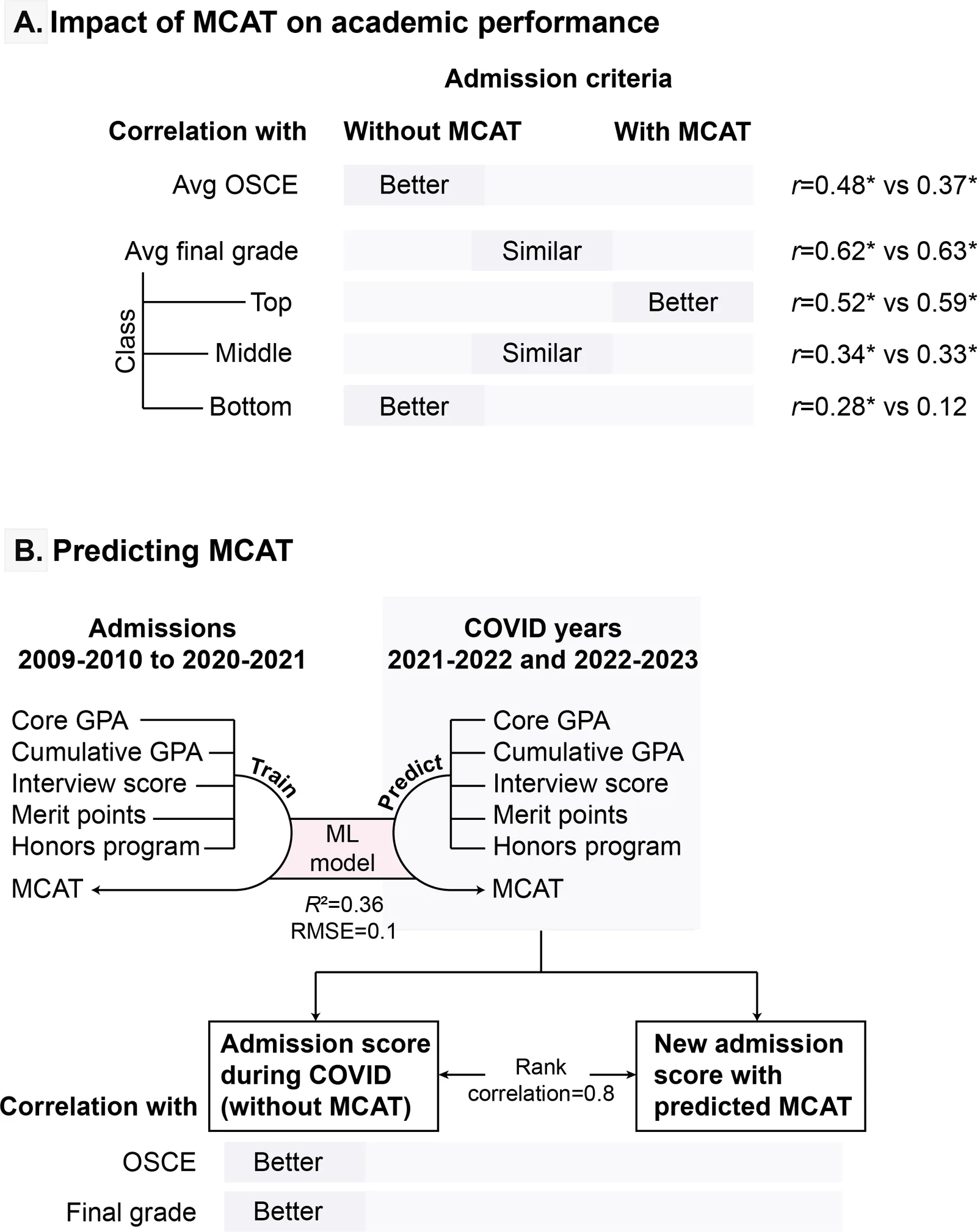
High-level summary of the results. (A) Comparison of correlations with academic performance of admission scores calculated with and without Medical College Admission Test (MCAT). (B) A predicted MCAT score was used to calculate an admission score during COVID years, and its impact on future academic performance was analyzed. *A statistically significant correlation (*P*<.05; Table 3). GPA: grade point average; ML: machine learning; OSCE: objective structured clinical examination; RMSE: root mean squared error.

Figure 5 summarizes the key findings across all analyses. Including the MCAT in the admissions score did not improve the prediction of final-year grades and was associated with worse prediction of OSCE grades. Furthermore, when we predicted MCAT scores from other admission criteria and incorporated it into the admission score, predictive performance was also reduced for both outcomes.

## Discussion

### Principal Findings

This study examined 2 related questions: the incremental validity of observed MCAT scores for predicting medical school performance (objective 1) and the feasibility and decision-impact of ML-predicted MCAT scores when testing is unavailable (objective 2). By addressing both the role of MCAT within holistic admissions and the potential for algorithmic substitution, the findings contribute to ongoing discussions regarding standardized testing in medical education and the use of data-driven tools in admission selection.

### MCAT and Academic Performance (Objective 1)

Our analysis indicates that within our institution and study population, the inclusion of MCAT scores did not substantially enhance the prediction of short-term academic outcomes beyond an already rigorous admissions process incorporating undergraduate GPA and structured interviews. Admissions composites generated during the temporary MCAT suspension showed associations with subsequent academic performance that were largely comparable to those observed under the traditional MCAT-inclusive model. Practically, this implies that uGPA plus structured interviews (and other holistic factors) can provide a robust basis for selection in the short term.

Our findings, however, should not be interpreted as evidence against the value of standardized testing altogether. Historically, the MCAT has served as a standardized comparator across heterogeneous educational backgrounds within the North American graduate-entry medical admissions model. Rather, our results suggest that the incremental contribution of MCAT scores may become smaller when admissions systems already incorporate multiple academic and nonacademic measures.

These findings should be interpreted alongside the broader literature. Large multisite studies and recent meta-analyses have consistently demonstrated that MCAT scores are moderately associated with medical school and licensing examination performance [7,8,29-31]. However, single-institution studies have also shown comparable early outcomes with MCAT-blinded admissions after controlling for uGPA [32]. Thus, while our results suggest that robust holistic processes can mitigate the short-term impact of MCAT suspension at our school, they should not be interpreted as demonstrating limited incremental value of the MCAT across medical schools. Institutional differences in applicant pools, admissions weightings, curricular structure, assessment methods, and resource availability may produce different outcomes elsewhere.

There are several reasons why our results failed to show an incremental value of the MCAT in predicting future performance. First, our holistic admissions process may already capture much of what the MCAT measures. Evidence suggests that when noncognitive assessments are robust, the incremental validity of standardized tests diminishes [18,33]. Second, our relatively small sample size (n=349) and single-institution design limit statistical power to detect small effect sizes and restrict generalizability. Finally, and most importantly, our correlations are not corrected for restriction of range, a limitation we address in detail below.

The finding that including the MCAT in admissions scoring weakened the prediction of clinical skills performance, as measured by the average OSCE grades in Med 1 and Med 2, is entirely consistent with existing literature demonstrating that the MCAT primarily predicts knowledge-based examination performance rather than clinical assessments. For example, Saguil et al [34] found no significant association between MCAT scores and OSCE performance, step 2 clinical skills subscores, or postgraduate year 1 program director evaluations, findings that have been supported by other reports [6,7,35,36].

This pattern is conceptually expected because the MCAT primarily assesses scientific knowledge and critical reasoning, whereas OSCEs evaluate communication, clinical reasoning, professionalism, and interpersonal skills [37,38], consistent with studies demonstrating stronger associations between MMIs and clinical performance [39,40], which may explain why the non-MCAT composite (with greater relative weight on interviews) showed stronger correlations with OSCE outcomes.

Another interesting, and unexpected, finding was that among students in the lower tertile of admission scores (where we would have expected the MCAT to strengthen prediction), the MCAT-inclusive composite was not significantly correlated with final grades, as opposed to the MCAT-excluded composite. This suggests that for students at the margin of admission decisions, where selection decisions are most difficult and consequential, MCAT scores may fail to add predictive value. Students selected with lower MCAT scores (bottom tertile) may have other compensatory (noncognitive) abilities that contribute to their future success.

### ML Model Performance (Objective 2)

The ML model showed limited success in predicting MCAT scores (*R*²=0.36, RMSE=10%), explaining only 36% of the variance despite incorporating cumulative and core GPAs, interview scores, merit points, and honors program participation.

Although an *R*² of 0.36 may be considered substantial in some applied settings [41], it is modest for predicting one standardized academic measure from other academic variables, suggesting that the MCAT captures information not fully reflected by GPA and interview performance. Nevertheless, even perfect prediction of MCAT scores would have limited impact in our setting, as our primary finding was that the MCAT itself added negligible incremental validity to admission decisions.

Incorporating predicted MCAT scores into the composite for the 2 cohorts admitted during the MCAT suspension produced rankings that were strongly aligned with the original admissions outcomes, yet it would have altered the admission of 10 students across the 2 years, with those students replaced by others from the previously rejected pool. Importantly, the model’s RMSE corresponds to an error of several MCAT points, large enough to shift rank order for applicants near the admissions cutoff. Accordingly, despite strong overall rank correlation, predicted MCAT values should not be treated as interchangeable substitutes for observed MCAT scores in high-stakes decision-making.

These findings do not reflect a flaw in the modeling strategy. We used a robust pipeline and an ensemble of linear and nonlinear learners, yet prediction error remained large enough to influence rank order near the admissions threshold. This likely reflects an inherent limitation of attempting to reconstruct a standardized test score from a limited set of admissions variables that were not designed to reproduce the MCAT at the individual level. In practice, while predicted MCAT scores may approximate population-level trends, they are not sufficiently precise for high-stakes individual ranking decisions.

These findings highlight an important limitation of current ML approaches. While ML may support tasks such as initial applicant screening [42], detecting biases in human decisions [43], or identifying patterns in large datasets [44], our results suggest that it is not sufficiently accurate to replace standardized test scores in high-stakes admissions decisions.

A key takeaway from this modeling strategy is the importance of considering multiple metrics for model performance. While *R*² is a commonly used measure, it does not provide a complete picture on its own. The RMSE, which reflects the model’s average error, should also be considered. Although there is no universally accepted threshold for RMSE, it should be interpreted based on the specific context.

### Limitations

This study has several important limitations that warrant consideration when interpreting our findings.

First, our findings come from one medical school with a specific admissions process, curriculum, and student population, which limits generalizability to other institutions and contexts. Consequently, external validity is limited. Other schools that rely more heavily on standardized testing, that use different interview formats (eg, interview calibration and rubric design), or that have distinct curricular or assessment systems may observe different incremental validity for the MCAT. Multi-institutional studies with variation in admissions models and outcome measures are required before recommending broader policy change. Second, our analyses were limited to admitted students, introducing restriction-of-range effects that likely reduced the magnitude of observed correlations relative to the full applicant pool. Because outcome data were unavailable for rejected applicants, formal correction for range restriction could not be performed. Therefore, our findings likely underestimate true population-level associations and should be interpreted cautiously when compared with range-corrected studies. This limitation likely affects both the MCAT and non-MCAT admissions models, although the extent of its impact cannot be determined from our data. Therefore, the comparative analyses should be interpreted cautiously. Third, we relied only on medical school grades and early OSCE scores (Med 1‐2 only) as performance indicators. We did not examine longer-term outcomes such as licensing examination performance (United States Medical Licensing Examination [USMLE] Steps), residency matching success, clinical performance in residency, or ultimate career trajectories. Fourth, we used Pearson correlations to assess predictive validity, which capture only linear relationships. Important nonlinear patterns or interaction effects may exist that our analytical approach did not detect. Finally, our admissions process evolved over the study period, particularly with MMI introduction in 2018 and weight adjustments. While we accounted for these changes analytically, the evolving nature of our process introduces some heterogeneity. Because of the relatively small sample size, we were unable to perform meaningful sensitivity analyses by time period (eg, pre-MMI vs post-MMI implementation), and temporal changes may therefore have influenced the observed associations. Additionally, the COVID-19 pandemic brought further potential confounders, such as remote or virtual interview formats and short-term curricular modifications, which may have affected interview scores, early OSCE experiences, and the relationships between admissions metrics and outcomes. These pandemic-related changes could therefore have contributed to greater variability in the pandemic cohorts and should be taken into account when generalizing our findings.

### Conclusions

In this single-institution study, we addressed 2 related questions: whether including MCAT scores adds incremental validity to admissions decisions and whether an ML model can substitute for the MCAT when testing is unavailable. Our findings suggest that when the MCAT is temporarily unavailable, a well-designed holistic admissions process emphasizing careful GPA review and interviews maintained short-term student quality. Accordingly, the value of this analysis lies less in proposing a new admissions tool than in demonstrating the current limitations of ML as a substitute for standardized testing in this context. Rather than attempting to recreate MCAT scores, efforts should focus on strengthening validated noncognitive assessments, particularly interviews such as the MMI, while maintaining individualized review of applicants near the admissions threshold. Institutions considering reduced reliance on the MCAT should validate such changes locally (eg, via retrospective simulations or pilot cohorts) and monitor medium-term and long-term outcomes. More broadly, within the context of this single-institution study, our findings support viewing standardized testing as one component of a comprehensive, evidence-informed admissions process, rather than as a standalone admission criterion. Further, multi-institutional studies are needed before broader conclusions regarding the incremental value of the MCAT can be drawn and to evaluate alternative approaches for MCAT estimation.

## Abbreviations

GPA: grade point average
LAU: Lebanese American University
LightGBM: light gradient boosting machine
MCAT: Medical College Admission Test
ML: machine learning
MMI: multiple mini-interview
OSCE: objective structured clinical examination
RMSE: root mean squared error
SOM: School of Medicine
uGPA: undergraduate grade point average
USMLE: United States Medical Licensing Examination
